# Correction: Modelling Influence and Opinion Evolution in Online Collective Behaviour

**DOI:** 10.1371/journal.pone.0230584

**Published:** 2020-03-12

**Authors:** The PLOS ONE Staff

S1 Dataset is omitted from the list of Supporting Information. It can be viewed below. The publisher apologizes for the error.

## Supporting information

S1 Dataset Supplementary data. (ZIP)
