# Supplementary material for: Modelling Influence and Opinion Evolution in Online Collective Behaviour
Source: PLoS One. 2016 Jun 23;11(6):e0157685. doi: 10.1371/journal.pone.0157685 (PMC4918933; doi:10.1371/journal.pone.0157685)
Supplement: S1 File — (PDF) [file pone.0157685.s001.pdf]

Supplementary Information
Modelling influence and opinion evolution in online collective behaviour

Corentin Vande Kerckhove, Samuel Martin, Pascal Gend, Peter J. Rentfrow, Julien M. Hendrickx, Vincent D. Blondel

A Derivation of the measure of unpredictability

A.1 Proof that η has zero mean

In section *Intrinsic unpredictability estimation*, we used that η has zero mean. This fact is proven in the sequel. Assume, to show a contradiction that $m = \mathbb{E}(\eta) \neq 0$. Denote $\tilde{x}_i(2) = f_i^1(x_i(1), x_{others}(1), picture)$. Then, we can show that $\tilde{x}_i(2) + m$ would be a better model for $x_i(2)$ than $\tilde{x}_i(2)$. This would contradict the definition of f_i^1 as the best model. Indeed, the expected square prediction error would be

$$\begin{aligned}\mathbb{E}\left((x_i(2) - (\tilde{x}_i(2) + m))^2\right) &= \mathbb{E}\left((x_i(2) - \tilde{x}_i(2))^2\right) - 2m\mathbb{E}\left(x_i(2) - \tilde{x}_i(2)\right) + m^2 \\ &= \mathbb{E}\left((x_i(2) - \tilde{x}_i(2))^2\right) - m^2 \\ &< \mathbb{E}\left((x_i(2) - \tilde{x}_i(2))^2\right),\end{aligned}$$

where we used the fact that $\eta = x_i(2) - \tilde{x}_i(2)$. The same reasoning allows to show that the prediction error $\bar{\eta}$ at round 3 also has zero mean.

A.2 Derivation of equation (3)

Equation (3) is derived using the following reasoning. The judgments made in two replicated games of a control experiment by a same participants are described as

$$\begin{aligned}x_i(2) &= f_i^1(x_i(1), x_{others}(1), picture) + \eta, \\ x_i'(2) &= f_i^1(x_i'(1), x_{others}'(1), picture) + \eta',\end{aligned}$$

where the prime notation is taken for judgments from the second replicated game and η and η' are two independent draws of the random intrinsic variation. By design, the set of judgments are all shifted by the same constant :

$$\begin{aligned}x_i'(1) &= x_i(1) + s, \\ x_{others}'(1) &= x_{others}(1) + s,\end{aligned}$$

where $s = x_i'(1) - x_i(1)$ is known. According the assumption made on function f_i^1,

$$\begin{aligned}x_i(2) &= \lambda g_i^1(x_i(1), x_{others}(1)) + (1-\lambda) h_i^1(picture) + \eta, \\ x_i'(2) &= \lambda g_i^1(x_i'(1), x_{others}'(1)) + (1-\lambda) h_i^1(picture) + \eta',\end{aligned}$$

the second round judgment made in the second replicate is then

$$x_i'(2) = \lambda\left(g_i^1(x_i(1), x_{others}(1)) + s\right) + (1-\lambda)h_i^1(picture) + \eta',$$

where the invariance by translation of g_i^1 was used. Taking the difference makes the unknown terms $g_i^1(x_i(1), x_{others}(1))$ and $h_i^1(picture)$ vanish to obtain

$$x_i'(2) - x_i(2) = \lambda s + \eta' - \eta. \tag{6}$$

Since η and η' have zero mean and are assumed to have equal variance, the theoretical variance of η is

$$\begin{aligned}
\mathbb{E}(\eta^2) &= \frac{1}{2}\left(\mathbb{E}(\eta^2) + \mathbb{E}(\eta'^2)\right) \\
&= \frac{1}{2}(\mathbb{E}(\eta'^2) - 2\mathbb{E}(\eta'\eta) + \mathbb{E}(\eta^2) + 2\mathbb{E}(\eta'\eta)) \\
&= \frac{1}{2}(\mathbb{E}(\eta'^2 - 2\eta'\eta + \eta^2)) + \mathbb{E}(\eta'\eta) \\
&= \frac{1}{2}\mathbb{E}((\eta' - \eta)^2) + \mathbb{E}(\eta'\eta).
\end{aligned} \tag{7}$$

Moreover η and η' are assumed to be independent with zero mean, *i.e.,* , $\mathbb{E}(\eta) = \mathbb{E}(\eta') = 0$, therefore, their covariance is null : $\mathbb{E}(\eta\eta') = \mathbb{E}(\eta)\mathbb{E}(\eta') = 0$. Consequently, $\mathbb{E}(\eta^2) = \frac{1}{2}\mathbb{E}((\eta' - \eta)^2)$ and using equation (6), the variance of η is empirically measured as the average of

$$\frac{1}{2}(x_i'(2) - x_i(2) - \lambda s)^2$$

over all repeated games and all participants. This corresponds to equation (3).

A.3 Discussion on the assumptions on η and η'

The only assumption used to derive equation (6) is that η and η' have the same variance and are independent for each participant. Since function f_i^1 is unknown, it is not possible to directly test these assumptions. However, since pairs of replicates in the control experiment are related to the same picture, it is unlikely that the covariance between η and η' would be negative. If the covariance was positive, the quantity given in equation (6) would become a lower bound on the unpredictability threshold, as shown through equation (7). Finally, if η and η' did not satisfy the assumption of equal variance, the quantity in equation (6) would still correspond to the average variance $\frac{1}{2}\left(\mathbb{E}(\eta^2) + \mathbb{E}(\eta'^2)\right)$ which also represents the average intrinsic unpredictability, as seen in equation (7).

B Circumstances of the wisdom of the crowd

The wisdom of the crowds may not always occur. The present section recalls one important hypothesis underlying the wisdom of the crowds. The hypothesis is then tested against the empirical data from the study. In the context of the present study, the wisdom of the crowd corresponds to the following fact: the mean opinion is most often *much* closer to the true answer than the individual

opinions are. Denoting $\bar{x}$ the mean of n opinions x_i and T the corresponding true answer, this is formally expressed as

$$|\bar{x} - T| << \frac{1}{n}\sum_{i=1}^{n}|x_i - T|, \quad (8)$$

where $<<$ stands for *significantly smaller than.* The wisdom of the crowd given by equation 8 does not always take place. It only occurs if the opinions x_i are distributed sufficiently symmetrically around the true answer. When the distribution is largely biased above or below the true answer, equation 8 fails to hold. To understand this fact, the group of individual is split in two : $i \in \mathcal{N}^+$ if $x_i(1) > T$ and $i \in \mathcal{N}^-$ if $x_i(1) < T$. Then, the distance of the mean opinion to truth rewrites as

$$\begin{aligned} |\bar{x} - T| &= \left| \left(\frac{1}{n}\sum_{i=1}^{n} x_i\right) - T \right| = \frac{1}{n}\left|\sum_{i=1}^{n}(x_i - T)\right| = \frac{1}{n}\left|\sum_{i\in\mathcal{N}^+}(x_i - T) + \sum_{i\in\mathcal{N}^-}(x_i - T)\right| \\ &= \frac{1}{n}\left|D^+ - D^-\right| \end{aligned}$$

where $D^+ = \sum_{i\in\mathcal{N}^+}|x_i - T| \geq 0$ is the contribution from opinions above truth and $D^- = \sum_{i\in\mathcal{N}^-}|x_i - T| \geq 0$ is the contribution from opinions below truth. Using these notation, the average distance to truth is $\frac{1}{n}\sum_{i=1}^{n}|x_i - T| = \frac{1}{n}(D^+ + D^-)$. As a consequence, the wisdom of the crowd described in equation 8 translates to

$$\left|D^+ - D^-\right| << (D^+ + D^-). \quad (9)$$

Two extreme cases are possible :

- **Perfect wisdom of the crowd :** opinions are homogeneously distributed around the true answer and $D^+ = D^-$ so that $|\bar{x}(1) - T| = 0$.
- **No wisdom of the crowd :** opinions either totally overestimate or totally underestimate the correct answer and either $D^- = 0$ or $D^+ = 0$, so that $|\bar{x} - T| = \frac{1}{n}\sum_{i=1}^{n}|x_i - T|$.

We now turn to the empirical data. Only the first round is discussed here because, in the subsequent rounds, the opinions are no longer independent, a criterion required for the wisdom of the crowd to occur. Fig A displays how opinions are distributed around the true value for the *gauging game* (A) and the *counting game* (B). Both distributions fall between the two extreme cases with most opinions underestimating the true value. However, the bias is more important in the *counting game* which explains that the wisdom of the crowd is more prominent in the *gauging game* in the first round. This explains the differences between mean opinion errors and individual errors observed in Fig 5.

C Testing the linearity of the consensus model

The consensus model (1) assumes that the opinion change $x_i(t+1) - x_i(t)$ grows linearly with the distance between $x_i(t)$ and the mean opinion $\bar{x}(t)$. This assumption is tested against the alternative

$$x_i(t+1) - x_i(t) = \beta_0 + \alpha_1(\bar{x}(t) - x_i(t))^\gamma$$

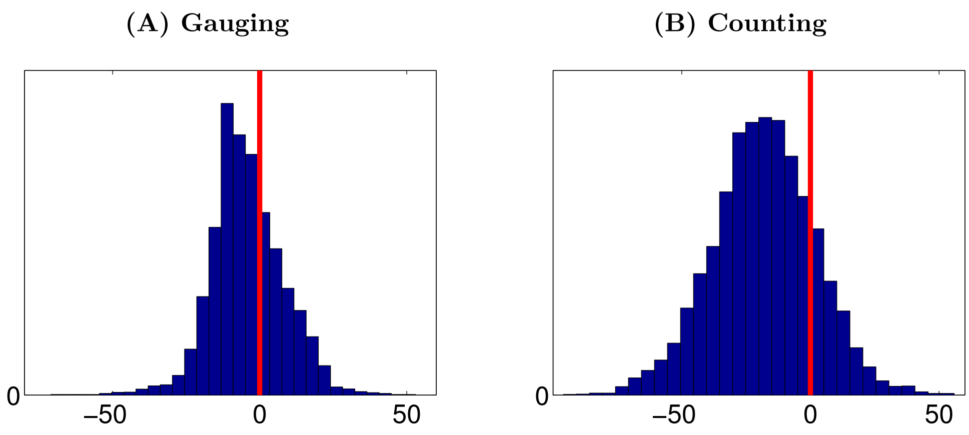

Figure A. Deviation of opinions to truth during the lone round 1. Red vertical lines split the histogram into the opinions contributing negatively to the distance between mean opinion and truth (left) and the opinions contributing positively to the distance between mean opinion and truth (right). (A) *gauging game*; (B) *counting game*.

with $\gamma \neq 1$. The numerical statistics values are reported for the opinion change between rounds 1 and 2 for the *gauging game*. The same conclusions hold for the *counting game* and for the opinion change between rounds 2 and 3. The linearity test provided in [66] applied to our data gives a statistics $P = -1.4\text{E}7$ with empirical variance $\text{var}(P) = 4\text{E}14$ so that we fail to reject the null hypothesis $\gamma = 1$ (p-val=0.5). Fig B displays the evolution $x_i(t+1) - x_i(t)$ against the distance to the mean $\bar{x}(t) - x_i(t)$ along with the result of the linear regression assuming $\gamma = 1$.

D Influenceability and personality

Is influenceability related to personality ? To answer this question, we required the participants to provide information regarding their personality, gender, highest level of education, and whether they were native English speaker. The questionnaire regarding personality comes from a piece of work by Gosling and Rentfrow [39] and was used to estimate the five general personality traits. The questionnaire page is reported in Fig F. For each of the five traits, the participants rated how well they feel in adequacy with a set of synonyms (rating $s \in \{1, \ldots, 7\}$) and with a set of antonyms (rating $a \in \{1, \ldots, 7\}$. This redundancy allows for testing the consistency of the answer of each participants. The participants who had a distance $(8 - a) - s$ too far away from 0 were discarded (threshold values were found using Iglewicz and Hoaglin method based on median absolute deviation [47]). Partial Pearson's linear correlations are first reported between the individual traits measured by the questionnaire (see table A). The correlation signs are found to be consistent with the related literature on the topic [67]. This indicates that our measure of the big five factors is trustworthy. Partial correlations are then provided to link the personal traits to influenceability. As shown in Table B, none of the measured personal traits is able to explain the variability in the influenceability parameter. The only exceptions concern gender and being English native speaker, with weak level of significance (p-*val* $\in [0.01, 0.05]$). However, these relations are consistent neither

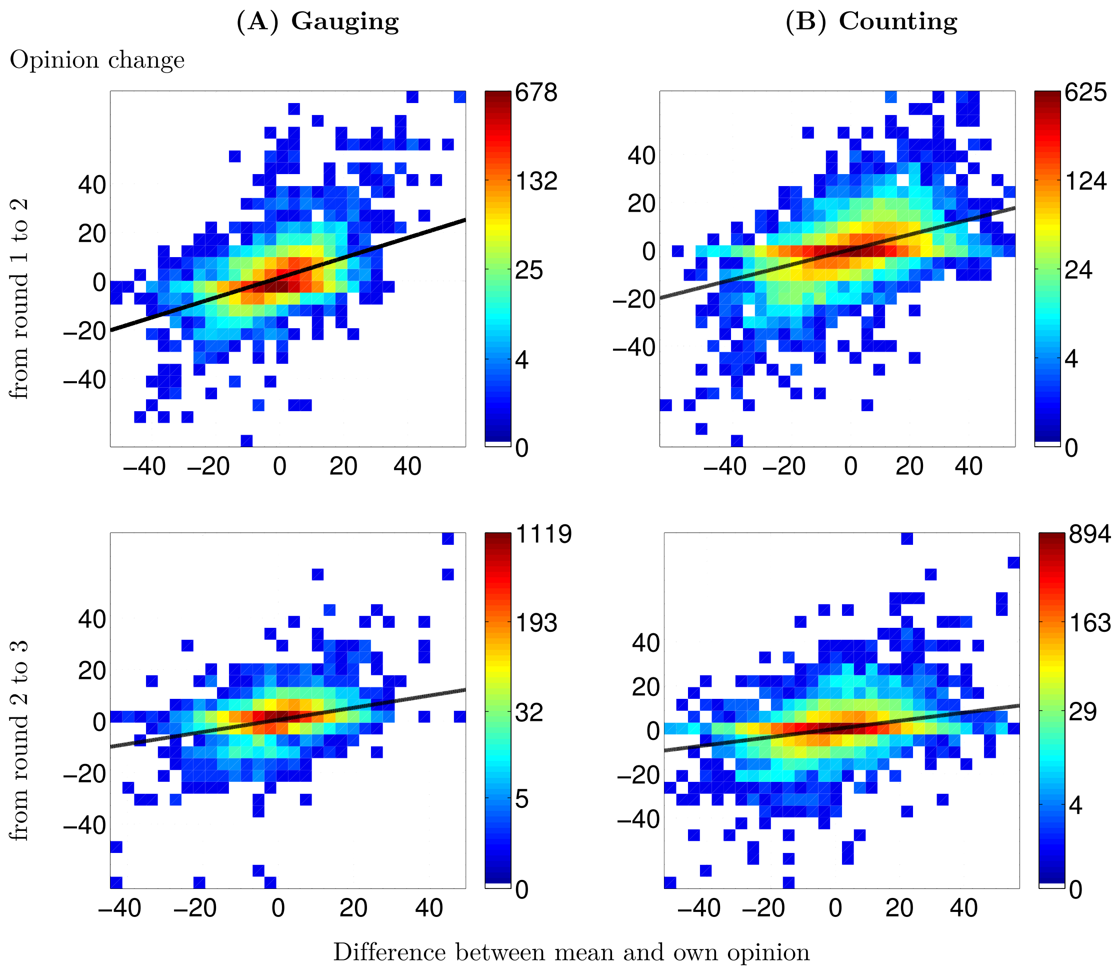

Figure B. Opinion change $x_i(t+1) - x_i(t)$ versus difference between mean and individual opinion $\bar{x}(t) - x_i(t)$. The color in each cell corresponds to the number of data points falling in the cell. The color scale is logarithmic. The black strait line represents the linear regression $x_i(t+1) - x_i(t) = \beta_0 + \alpha(t)(\bar{x}(t) - x_i(t))$. Top : opinion change from round 1 to 2 ; bottom : opinion change from round 2 to 3. (A) *gauging game*; (B) *counting game*.

between types of tasks nor over rounds, so that they cannot be trusted. We conclude that the big five personality factors and the other measured individual traits are not relevant to explain the influenceability parameter. Finding appropriate individual traits to explain the influenceability remains an open question.

(A) Participants from the *counting game*

	C	E	A	N	Gen	Eng	Edu
O	0.45***	0.38***	0.31***	-0.35***	0.03	-0.08	0.09
C		0.11	0.29***	-0.41***	-0.02	-0.06	0.29***
E			0.03	-0.15*	0.02	-0.05	0
A				-0.48***	0.11	-0.16*	0.15*
N					0.2**	0.08	-0.23***
Gen						0	0.01
Eng							-0.12

(B) Participants from the *gauging game*

	C	E	A	N	Gen	Eng	Edu
O	0.37***	0.24***	0.29***	-0.4***	0.06	0.15*	0.16**
C		0.2***	0.22***	-0.29***	0.15**	0.12*	0.11
E			-0.12*	-0.12*	0.04	0.04	-0.03
A				-0.34***	0.01	-0.07	0.08
N					0.18**	-0.02	-0.16**
Gen						-0.03	0
Eng							-0.02

Table A. Partial Pearson's linear correlations among the big five factors of personality (O: openness, C : calmness, E : extroversion, A : agreeableness, N : neuroticism), gender (Gen), native English speaker (Eng) and highest level of education (Edu). Significance : *p-val< 0.05, **p-val< 0.01,***p-val< 0.001.

	(A) Gauging		(B) Counting	
	$\alpha(1)$	$\alpha(2)$	$\alpha(1)$	$\alpha(2)$
O	-0.1	-0.04	0.07	-0.05
C	0.08	-0.06	-0.05	-0.09
E	-0.1	0.02	-0.01	-0.01
A	0	-0.02	0.1	0.07
N	-0.06	0.03	-0.1	0.01
Gen	-0.14*	-0.05	0.03	0.12*
Eng	-0.09	0.02	-0.03	-0.12*
Edu	0.05	-0.1	-0.04	-0.1

Table B. Partial Pearson's linear correlations linking influenceability to the big five factors of personality (O: openness, C : calmness, E : extroversion, A : agreeableness, N : neuroticism), gendre (Gen), native English speaker (Eng) and highest level of education (Edu). Significance : *p-val< 0.05, **p-val< 0.01,***p-val< 0.001.

References

66. Niermann S. Testing for linearity in simple regression models. AStA Advances in Statistical Analysis. 2007;91(2):129–139.

67. Van der Linden D, te Nijenhuis J, Bakker AB. The general factor of personality: A meta-analysis of Big Five intercorrelations and a criterion-related validity study. Journal of research in personality. 2010;44(3):315–327.

E Additional figures for prediction accuracy

E.1 Confidence intervals for prediction errors

Fig C displays error bars for 95% confidence interval of the RMSEs. This figure reveals that the two methods depending on training set size do not perform significantly better than the consensus model with one couple of typical influenceabilities, even for large training set sizes. This is an argument to favor the model in which the whole population has a unique couple of influenceabilities $(\alpha(1), \alpha(2))$.

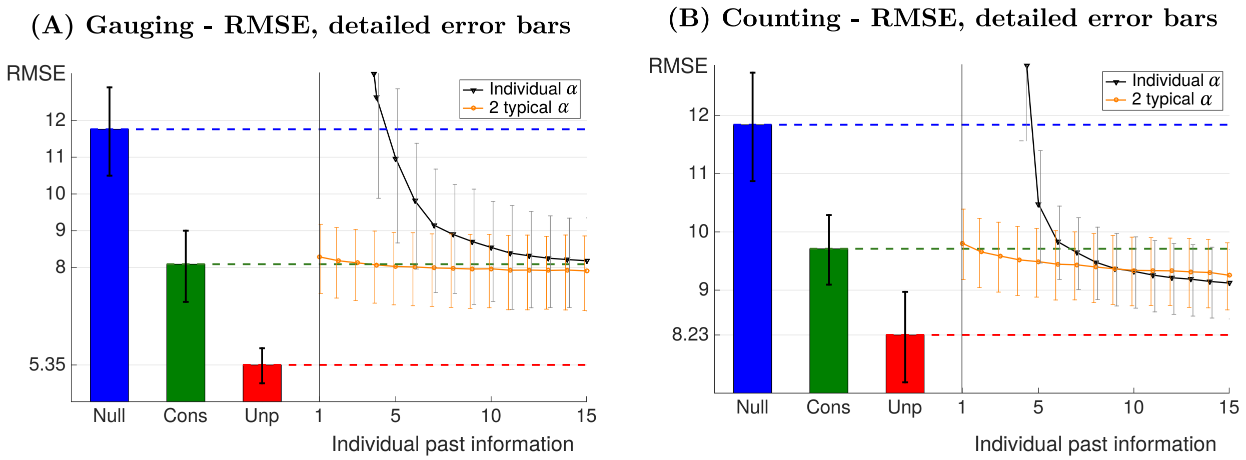

Figure C. Root mean square error (*RMSE*) of the predictions with detailed error bars for the final round.

E.2 Prediction accuracy in terms of Mean Absolute Errors

Measuring prediction accuracy in terms of MAEs may appear more intuitive for comparing prediction methods. Fig D. assesses the models using an absolute linear scale, where the errors are deliberately unscaled for the *counting game*. The prediction methods rank equally when measured in terms of MAE or RMSE. Notice that, due to nonlinear relation between RMSE and MAE, on this alternative scale, the consensus models errors are now closer from the null model than from the unpredictability error. For comparison, recall that for the *gauging game*, the judgments range between 0 and 100 while they range between 0 and 500 for the *counting game*.

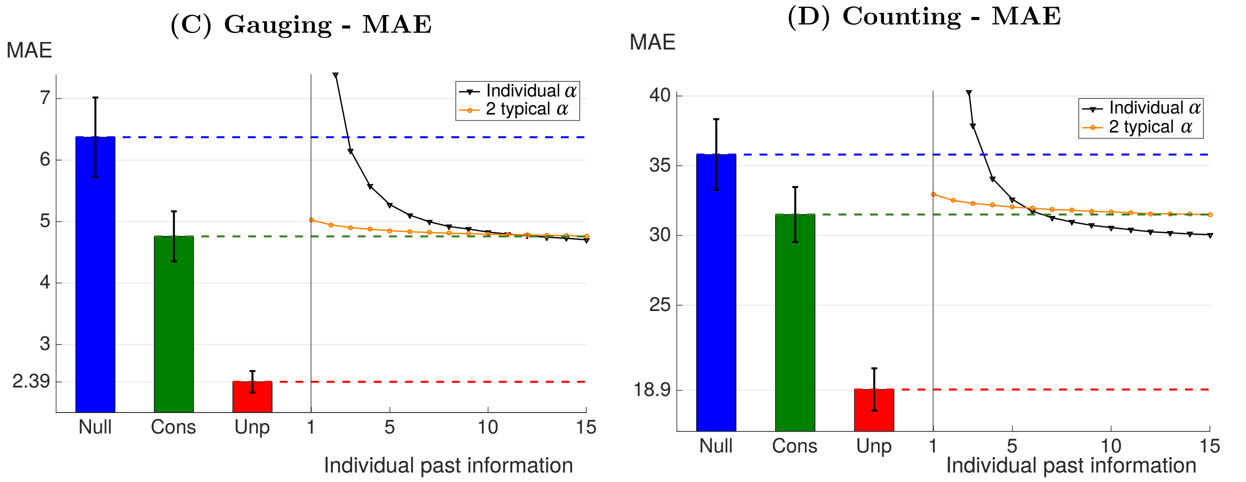

Figure D. Mean absolute error (*MAE*) of the predictions (unscaled) for the final round.

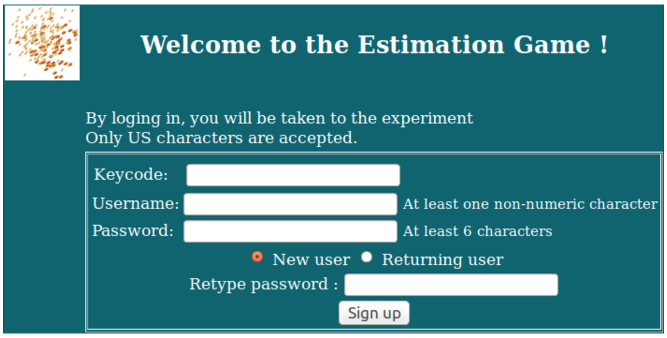

Welcome to the Estimation Game !

By loging in, you will be taken to the experiment
Only US characters are accepted.

Keycode:
Username: At least one non-numeric character
Password: At least 6 characters
New user Returning user
Retype password :
Sign up

Figure E. Login page.

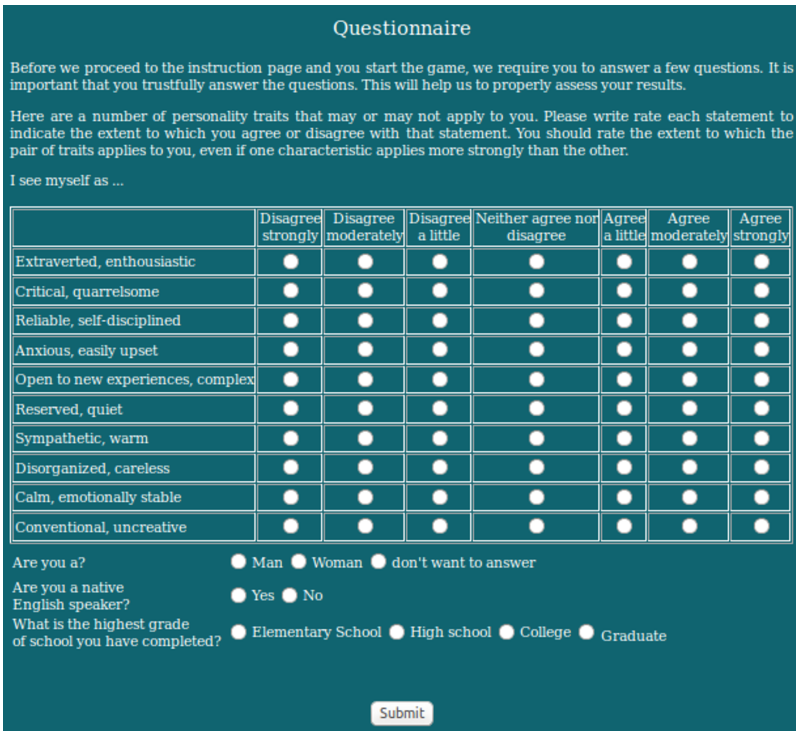

Questionnaire

Before we proceed to the instruction page and you start the game, we require you to answer a few questions. It is important that you trustfully answer the questions. This will help us to properly assess your results.

Here are a number of personality traits that may or may not apply to you. Please write rate each statement to indicate the extent to which you agree or disagree with that statement. You should rate the extent to which the pair of traits applies to you, even if one characteristic applies more strongly than the other.

I see myself as ...

	Disagree strongly	Disagree moderately	Disagree a little	Neither agree nor disagree	Agree a little	Agree moderately	Agree strongly
Extraverted, enthousiastic							
Critical, quarrelsome							
Reliable, self-disciplined							
Anxious, easily upset							
Open to new experiences, complex							
Reserved, quiet							
Sympathetic, warm							
Disorganized, careless							
Calm, emotionally stable							
Conventional, uncreative							

Are you a? Man Woman don't want to answer
Are you a native English speaker? Yes No
What is the highest grade of school you have completed? Elementary School High school College Graduate

Submit

Figure F. Questionnaire form.

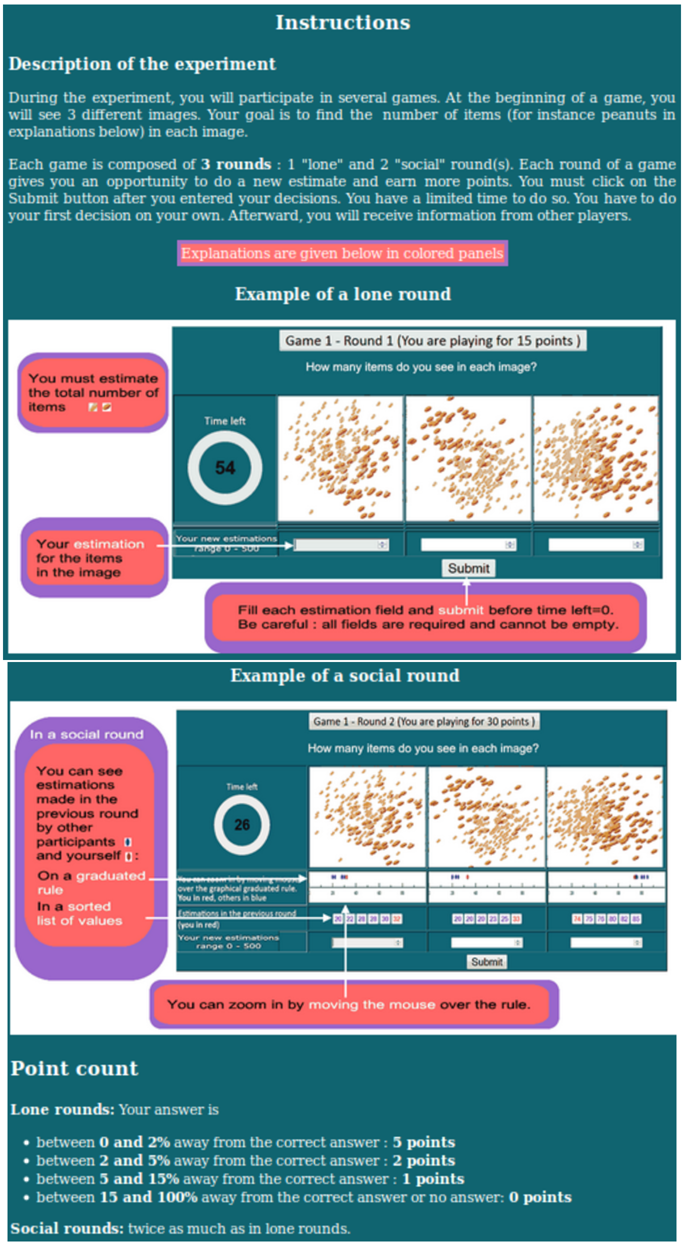

Instructions

Description of the experiment

During the experiment, you will participate in several games. At the beginning of a game, you will see 3 different images. Your goal is to find the number of items (for instance peanuts in explanations below) in each image.

Each game is composed of **3 rounds** : 1 "lone" and 2 "social" round(s). Each round of a game gives you an opportunity to do a new estimate and earn more points. You must click on the Submit button after you entered your decisions. You have a limited time to do so. You have to do your first decision on your own. Afterward, you will receive information from other players.

Explanations are given below in colored panels

Example of a lone round

Game 1 - Round 1 (You are playing for 15 points)
How many items do you see in each image?
Time left
54
You must estimate the total number of items
Your new estimations range 0 - 500
Your estimation for the items in the image
Submit
Fill each estimation field and submit before time left=0.
Be careful : all fields are required and cannot be empty.

Example of a social round

Game 1 - Round 2 (You are playing for 30 points)
How many items do you see in each image?
Time left
26
In a social round
You can see estimations made in the previous round by other participants and yourself :
On a graduated rule
In a sorted list of values
over the graphical graduated rule.
You in red, others in blue
Estimations in the previous round (you in red)
Your new estimations range 0 - 500
Submit
You can zoom in by moving the mouse over the rule.

Point count

Lone rounds: Your answer is

- between **0 and 2%** away from the correct answer : **5 points**
- between **2 and 5%** away from the correct answer : **2 points**
- between **5 and 15%** away from the correct answer : **1 points**
- between **15 and 100%** away from the correct answer or no answer: **0 points**

Social rounds: twice as much as in lone rounds.

Figure G. Instruction page.

(A)

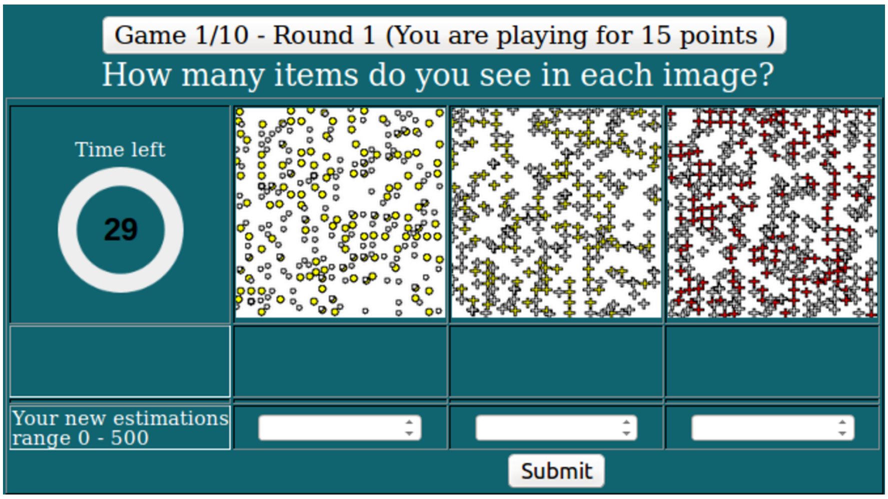

(B)

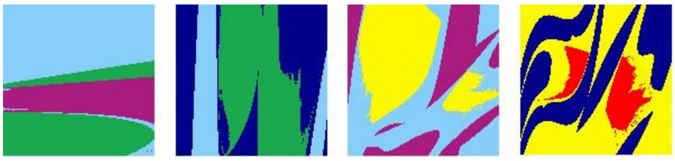

Figure H. (A) Interface of the first round for the *counting game*, played alone. (B) Instances of pictures for the *gauging game*.

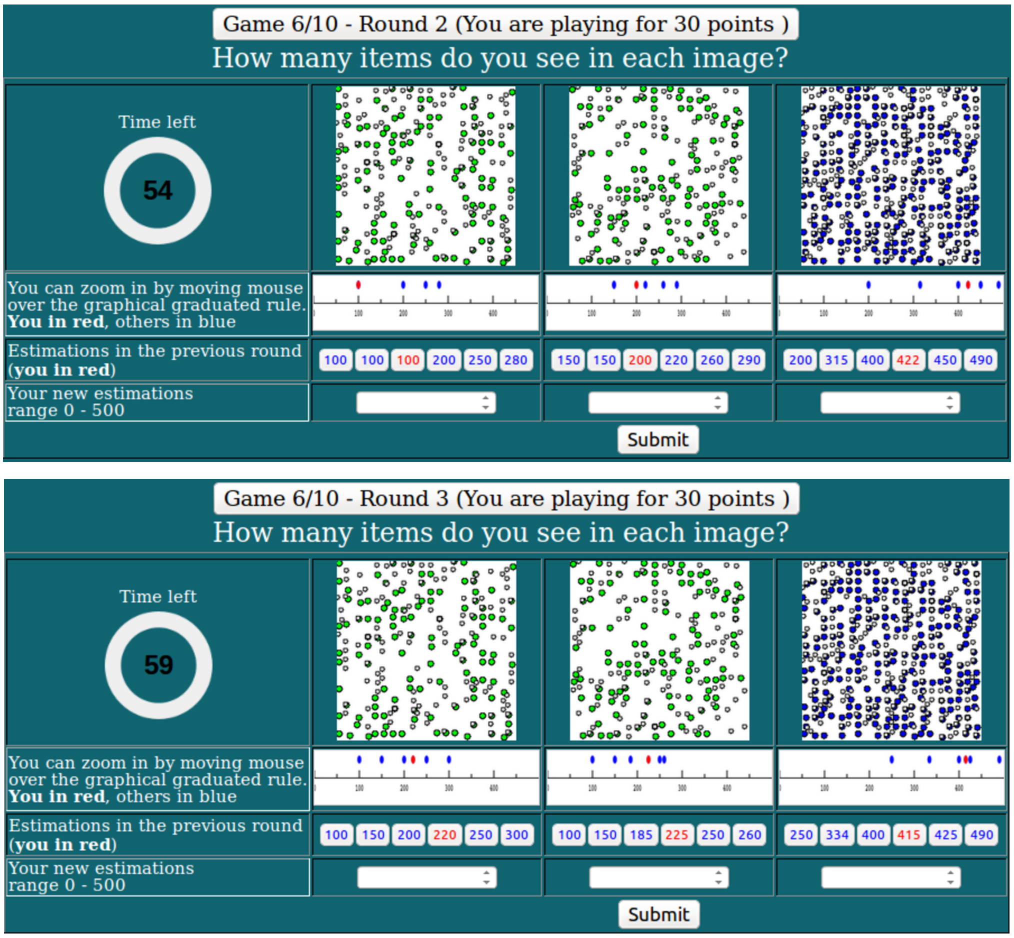

Figure I. Interface of the second and third rounds for the *counting game*, social rounds

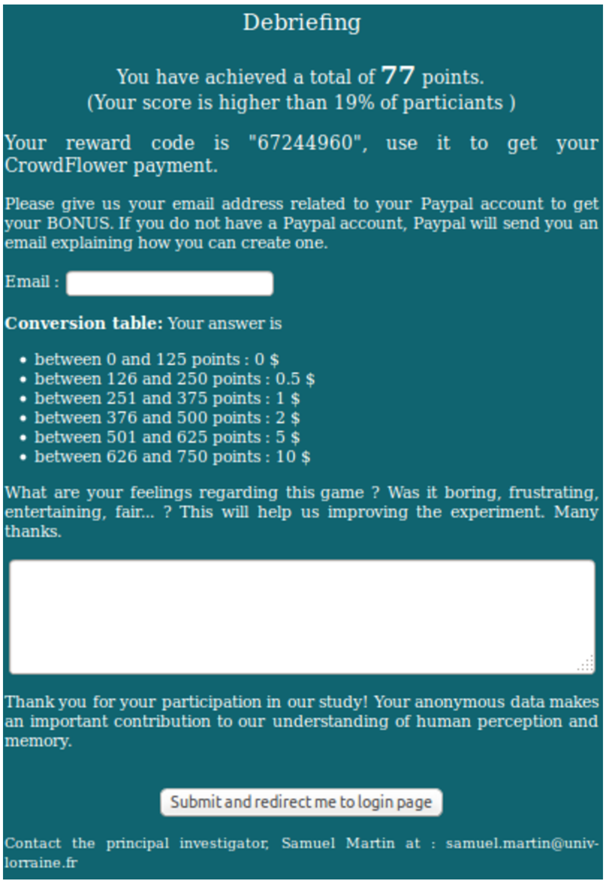

Figure J. Debrief page.
